# Effect of Hepatitis C Infection on HIV-Induced Apoptosis

**DOI:** 10.1371/journal.pone.0075921

**Published:** 2013-10-01

**Authors:** Tomasz Laskus, Karen V. Kibler, Marcin Chmielewski, Jeffrey Wilkinson, Debra Adair, Andrzej Horban, Grzegorz Stańczak, Marek Radkowski

**Affiliations:** 1 Department of Immunopathology, Warsaw Medical University, Warsaw, Poland; 2 The Biodesign Institute at ASU, Arizona State University, Phoenix, Arizona, United States of America; 3 St. Joseph’s Hospital and Medical Center, Phoenix, Arizona, United States of America; 4 Municipal Hospital for Infectious Diseases, Warsaw, Poland; University of North Carolina School of Medicine, United States of America

## Abstract

**Background:**

Hepatitis C virus (HCV) coinfection was reported to negatively affect HIV disease and HIV infection has a deleterious effect on HCV-related liver disease. However, despite common occurrence of HCV/HIV coinfection little is known about the mechanisms of interactions between the two viruses.

**Methods:**

We studied CD4+ and CD8+ T cell and CD19+ B cell apoptosis in 104 HIV-positive patients (56 were also HCV-positive) and in 22 HCV/HIV-coinfected patients treated for chronic hepatitis C with pegylated interferon and ribavirin. We also analyzed HCV/HIV coinfection in a Daudi B-cell line expressing CD4 and susceptible to both HCV and HIV infection. Apoptosis was measured by AnnexinV staining.

**Results:**

HCV/HIV coinfected patients had lower CD4+ and CD8+ T cell apoptosis and higher CD19+ B cell apoptosis than those with HIV monoinfection. Furthermore, anti-HCV treatment of HCV/HIV coinfected patients was followed by an increase of CD4+ and CD8+ T cell apoptosis and a decrease of CD19+ B cell apoptosis. In the Daudi CD4+ cell line, presence of HCV infection facilitated HIV replication, however, decreased the rate of HIV-related cell death.

**Conclusion:**

In HCV/HIV coinfected patients T-cells were found to be destroyed at a slower rate than in HIV monoinfected patients. These results suggest that HCV is a molecular-level determinant in HIV disease.

## Background

Hepatitis C virus (HCV) coinfection is very common among HIV-positive patients. Today up to 30% of all HIV-infected patients and as many as 85% of HIV-infected intravenous drug users (IDUs) are also HCV-positive [1]. HIV infection has a deleterious effect on HCV-related liver disease and due to falling AIDS mortality rates in the HAART era, liver disease has become the leading cause of death among HIV-infected patients [2–4]. These negative effects could be attributed to increased HCV replication in the setting of HIV-related immunosuppression, and some studies found close association between low CD4 cell count and the development of fibrosis and cirrhosis among coinfected patients [5,6]. However, there is some evidence that HIV may increase HCV replication more directly. Thus, primary HIV infection among HCV-positive patients is accompanied by a burst of HCV replication [7] and HCV RNA serum loads are more closely correlated with HIV RNA levels than with CD4+ cells [8]. It was also reported that concomitant or antecedent HIV infection may facilitate HCV replication in vitro [9]. Accelerated liver disease in HIV/HCV coinfection may also be the result of direct viral interactions as it was found that envelope proteins of both viruses may act collaboratively in injuring uninfected hepatocytes through an ‘innocent bystander’ mechanism [10].

However, HCV-HIV interactions are bidirectional as there is emerging evidence that HCV could negatively affect HIV disease. In several cohort studies HIV/HCV co-infected patients were found to be more likely to progress to AIDS and to have slower CD4 cell recovery on HAART, than those infected with HIV only [11–13].

Despite common occurrence of HCV/HIV coinfection, little is known about biological interactions between the two viruses. The current study provides evidence that HCV coinfection may affect HIV-related cell death both *in vivo* and *in vitro*.

## Methods

### Ethics Statement

The study was assessed and approved by the Internal Review Board at the Warsaw Medical University (ref No KBO/1/010) and each patient provided written consent.

### Patients

Two different groups of patients were studied. The first was composed of consecutive HIV-positive patients presenting for clinical outpatient care at the Municipal Hospital for Infectious Diseases, Warsaw, Poland. The inclusion criteria were: age above 18 years, willingness to participate in the study, and no advanced HIV disease (CD4+ cell count > 200 per cubic millimeter). Altogether, 56 were HCV-infected (anti-HCV and HCV RNA positive), and 48 patients were anti-HCV negative. Forty-five patients were on HAART and 41 had a history of intravenous drug use (IDU).

The second group was composed of 22 HIV/HCV-coinfected patients undergoing antiviral therapy for chronic hepatitis C. Treatment protocol consisted of Pegylated Interferon (Pegintron; Schering Plough) 1.5 µg/kg weekly and Ribavirin (Rebetol; Schering Plough) 800 mg/day for genotype 3 and 1000 mg/day for genotypes 1 and 4. All had compensated liver disease.

HIV RNA in serum was quantified by ABBOTT, Real Time PCR HIV CE (Abbott Molecular Inc., Des Plaines, IL); HCV RNA was quantified by ABBOTT, Real Time PCR HCV CE (Abbott Molecular, Inc).

### Apoptosis Assay of patients’ cells

PBMCs were isolated from blood by centrifugation over density gradient. Cells were washed 3 times with PBS (pH 7.4), suspended in RPMI 1640 medium containing 10% FBS, and incubated in plastic 6-well cell culture dish (Costar), at a final concentration of 10^6^ cells/ml. After incubation at 37 °C for 24 hours, CD4+, CD8+ and CD19+ cells were assessed for their sensitivity to spontaneous apoptosis by annexin V binding. Accordingly, cultured cells were divided into 3 parts and double stained with a fluorescein isothiocyanate-conjugated monoclonal antibody to annexin V (Annexin V kit, BD) and one of the phycoerythrin-conjugated monoclonal antibodies to CD4, CD8, and CD19 (BD Sciences). Cells were washed and fixed according to manufacturer’s procedure and immediately analyzed on a FACScalibur flow cytometer using Cell Quest software. For each sample, 10^4^ events were acquired. Propidium iodide (PI) positive cells were excluded from the analysis to differentiate between apoptotic and dead cells.

### Cell Line

Daudi-CD4+ cells, B-cell line transduced to express CD4, was a kind gift of Dr. Olivier Schwartz [14]. Daudi cells were chosen since they are susceptible to HCV infection *in vitro* [15] and B cells are commonly infected *in vivo* [16]. This line was maintained in RPMI-1640 supplemented with 10% fetal bovine serum (FBS). Sera used for *in vitro* infection experiments were collected from 7 chronic hepatitis C patients infected with genotype 1b. Viral loads were: P1 = 2.2 x 10^6^ IU/ml; P2 = 1.8 x 10^6^ IU/ml; P3 = 5 x 10^5^ IU/ml; P4 = 8.8 x 10^5^ IU/ml; P5 = 6.0 x 10^5^ IU/ml; 1W = 1.3 x 10^7^ IU/ml; 5W = 1.5 x 10^8^ IU/ml. Control sera (N1-N5) were collected from healthy donors and were negative for anti-HCV, anti-HIV, anti-HBc, and HGV RNA.

### Viral infections

Cells were split 24 hours prior to infection (Day -1). On Day 0, cells were counted, pelleted, and resuspended at a density of 5 x 10^5^ cells per ml in RPMI-1640 (10% FBS). Cells were then either infected with pNL4-3 (gift of K.T. Jeang, NIH) or mock infected for two hours at 37°C. Following the incubation, cells were pelleted, washed in complete RPMI-1640, and resuspended at a density of 5 x 10^5^ cells per ml. For incubations with patient serum, cells were pelleted (Day 1), washed with RPMI-1640 containing no serum, and then incubated with RPMI-1640 containing 10% human HCV-positive serum, or with RPMI-1640 containing 10% human HCV-negative serum, or with medium containing only 10% FBS. The infection was done at 37°C for 4 hours. Cells were then pelleted, washed in RPMI-1640 (no serum) and resuspended in complete RPMI-1640 plus 10% FBS.

Cells were counted each day beginning with Day 2, pelleted (supernatant was removed and set aside), and resuspended at a density of 5 x 10^5^ cells per ml in medium comprised of equal parts set-aside supernatant (to maintain HIV presence) and fresh medium. Samples of supernatants were stored at -20° for later use in HIV assays.

### Reverse Transcriptase (RT) Assay

HIV was quantitated by RT assay as previously described [17]: briefly, 10 µl supernatant was added to 40 µl cocktail (50 mM Tris Cl, pH 7.8, 63 mM KCl, 4.2 mM MgCl_2_, 0.08% Nonidet P-40, 0.85 mM EDTA, 4.2 µg/ml polyA, 0.13 µg/ml oligo dT, 4mM DTT, 2 µl/ml [^32^P]TTP) and incubated for 2 hours at 37°C; 5 µl of the reaction mix was spotted onto DEAE paper, fixed 2x in 2X SCC for 30 minutes, and placed on x-ray film. Arbitrary units were quantitated by densitometry reading of the film with Image Quant 5.2 software (Bio-Rad).

### Apoptosis Assay of Daudi cells

To measure apoptosis, 2 x 10^6^ cells were pelleted, washed 2X in PBS, resuspended in binding buffer (BD Sciences), and stained with AnnexinV-FITC and propidium iodide. Following staining, cells were washed 2X in binding buffer, resuspended in 4% paraformaldehyde (Electron Microscopy Sciences) in PBS, and fixation was continued for 2-3 hours. Data was captured with a Becton Dickinson FACScan, and analyzed with CellQuest software (BD, Mountain View). For each sample, 10^4^ events were acquired.

### Statistical Analysis

Continuous variables were compared with Mann-Whitney *U* test. Spearman’s coefficient was used to calculate correlations. *P* ≤ 0.05 was considered to be statistically significant.

## Results

### Apoptosis in HCV/HIV coinfected patients

We analyzed 104 consecutive HIV-positive patients: 56 were HCV positive and 48 patients were anti-HCV negative. Some characteristics of these patients are presented in Table 1.

**Table 1:** Characteristics of HIV/HCV coinfected patients as compared to HIV-monoinfected patients

*Characteristic*	*HIV+/HCV+ (n = 56)*	*HIV+/HCV- (n = 48)*	*Statistical Significance*
Age (yrs)	36.0 ± 1.3	43.6 ± 1.6	< 0.001
No. (%) of Women	15 (27)	4 (8)	0.021
No. (%) History of IDU	40 (71)	1 (2)	< 0.001
ALT (IU/L)	71.8 ± 6.5	60.2 ± 14.9	< 0.001
No. (%) on HAART	23 (41)	22 (46)	NS
HIV RNA in serum, IU/ml (x 10^4^)	2.38 ±0.62	0.99 ± 0.32	NS
CD4+ cell count (cells/mm^3^)	430 ± 29	464 ± 37	NS
% of apoptotic CD4+ T cells	5.45 ± 0.59	7.35 ± 0.76	0.039
% of apoptotic CD8+ T cells	12.07 ± 1.0	15.46 ± 1.2	0.049
% of apoptotic CD19+ cells	7.41 ± 1.4	4.80 ± 0.7	0.22

Continuous variables are reported as mean ± SEM; Apoptotic cells are those binding to Annexin V

Analysis of flow cytometry data revealed that among HIV/HCV-coinfected patients, CD4+ and CD8+ T cell apoptosis (Annexin V positive, PI negative) was lower than in patients infected with HIV only (Table 1). In the case of CD19+ cells, apoptosis was higher among dually-infected patients, although this difference did not reach statistical significance. There was no statistically significant correlation between CD4+ cell count or HIV RNA load and CD4+, CD8+, or CD19+ apoptosis in either HCV-positive or HCV-negative group. Similarly, there was no correlation between the HCV RNA load in serum and apoptosis in any of the analyzed cell populations.

While both groups did not differ with respect to CD4+ cell count, HIV viral load, or the number of patients on HAART, HIV/HCV-coinfected patients were significantly younger and most were IDUs, whereas among HIV-monoinfected patients history of IDU was rare. Because of the skewed distribution of IDU among our patients, statistical adjustment for this factor was not possible.

To mitigate the effect of the above confounding factors, we studied a group of HIV/HCV-coinfected patients undergoing treatment for chronic hepatitis C. We hypothesized that if HCV infection was indeed inhibitory to HIV-related apoptosis, drug-induced inhibition of HCV replication would be followed by subsequent increase in the level of apoptosis. Some basic characteristics of these patients are presented in Table 2. All patients had history of IDU but were required to remain abstinent for at least 6 months prior to the study. Three were on methadone treatment, 6 had abused alcohol, but were abstinent for 6 months or longer. The most common infecting genotype was type 1b found in 8 patients, followed by genotypes 3 and 4c/4d, which were found in 6 and 7 patients, respectively. In one patient the genotype was identified as 1a.

**Table 2:** Characteristics of 22 HIV/HCV coinfected patients undergoing treatment with Pegylated interferon and Ribavirin.

*Parameter*	*HIV+/HCV+ N=22*
Age (yrs)	34.5 (22-56)
Gender F (%)	7 (32)
History of IDU (%)	22 (100)
Alcohol abuse (%)	6 (27)
ALT (IU/L)	62 (21-522)
HAART therapy (%)	3 (14)
HCV RNA in plasma (log_10_)	5.87 (3.16-7.03)
CD4 counts (cells/mm^3^)	467 (324-885)
Genotype 1a (%)	1 (5)
1b	8 (36)
3a	7 (32)
4c/4d	6 (27)

Continuous variables are reported as median (range)

**Figure 1:**
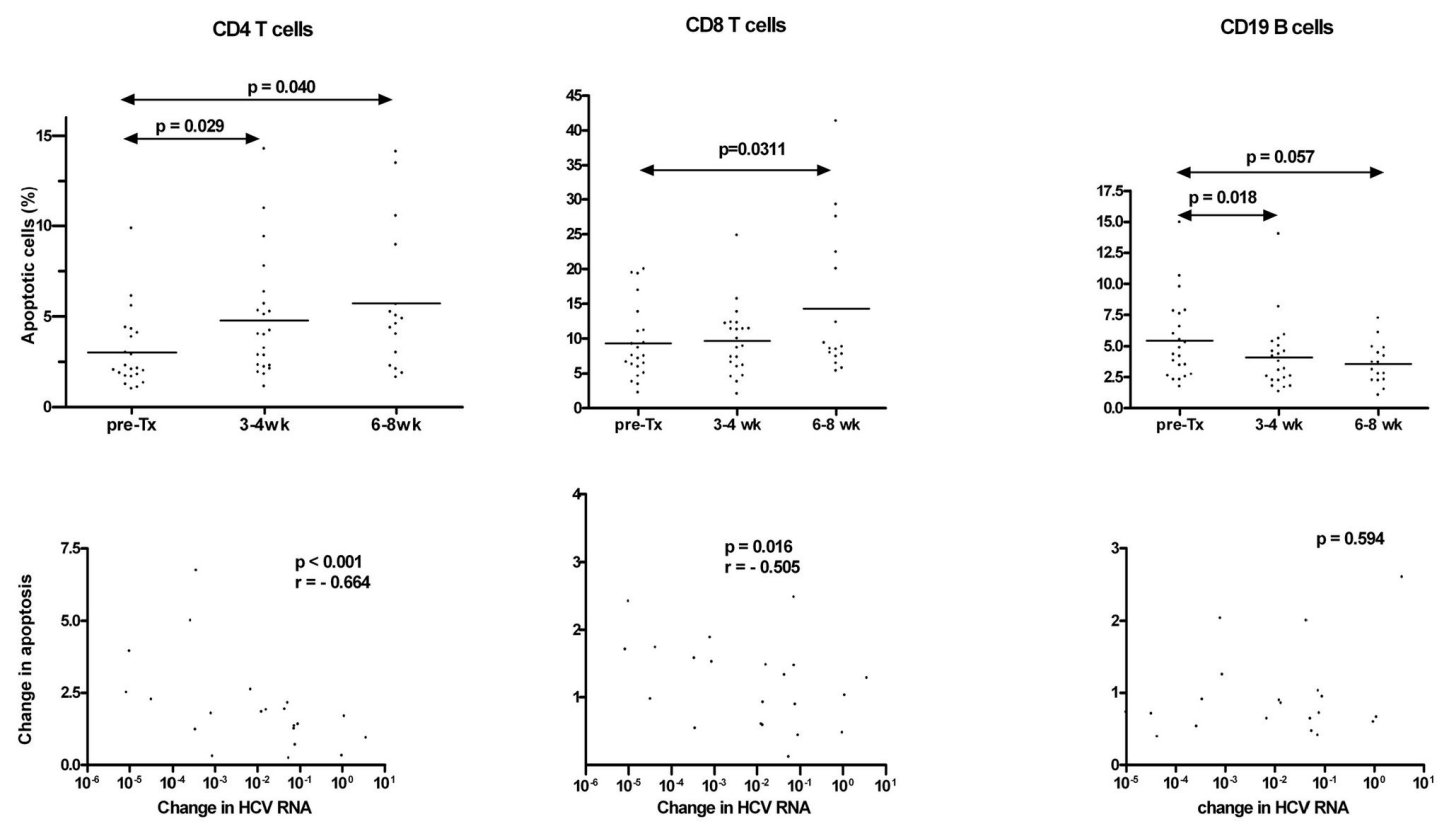
Changes in CD4+, CD8+ and CD19+ cells apoptosis in 22 HCV/HIV coinfected patients treated with pegylated interferon and ribavirin. Apoptosis was assessed by Annexin V staining and flow cytometry. Patients were tested right before initiation of treatment and after 3-4 weeks. In 16 patients testing was repeated 6-8 weeks from the initiation of treatment. Apoptosis of CD4+ and CD8+ T cells increased during therapy, while apoptosis of CD19+ B cells decreased.

Apoptosis was analyzed right before the initiation of treatment and at 3-4 weeks. In 16 patients a third analysis was done 6-8 weeks from the beginning of treatment. As seen in Figure 1, initiation of treatment resulted in an increase in apoptosis of CD4+ and CD8+ cells, while apoptosis of CD19+ cells decreased. Furthermore, drop in HCV RNA load correlated with increase in apoptosis of CD4+ (r=0.66; p< 0.001) and CD8+ cells (r=0.51; p=0.016).

### Apoptosis in Daudi CD4+ cells

**Figure 2:**
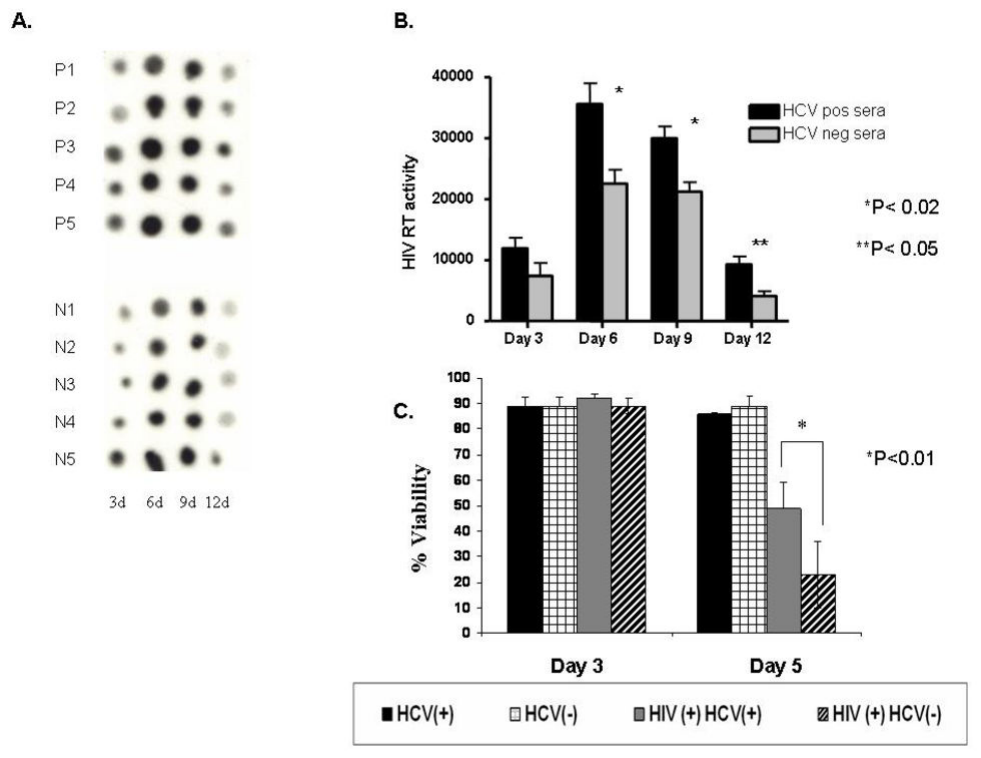
Infection of Daudi-CD4 cells with HIV. (A) Cells were infected with HIV (pNL4-3) and incubated with either HCV-positive patient serum (P1-P5) or HCV-negative serum (N1-N5) as described in Materials and Methods. Supernatant was harvested on days 3, 6, 9, and 12 and assayed for RT activity. Assay samples were spotted onto DEAE paper and visualized with autoradiography. (B) RT assay results quantified using densitometry. HIV RT activity is measured in arbitrary units. (C) To measure apoptosis resulting from exposure to HIV and HCV, cells were mock HIV-infected or infected with HIV then incubated with patient serum either positive or negative for HCV On days 3 and 5, cells were harvested, stained with annexinV (AV) and propidium iodide (PI), fixed, and measured for viability by flow cytometry. Viability was determined by the percentage of cells negative for both AV and PI. This experiment used 6 HCV-positive and 5 HCV-negative patient sera. Shown are the mean values ± SEM.

We first sought to determine if incubation with HCV-positive patient serum would have any effect on HIV particle production. We cultured cells in the absence of viral infection, or infected with HIV only, or infected with HIV followed by incubation with HCV-negative patient serum, or infected with HIV followed by incubation with HCV-positive patient serum. After exposure to HCV-positive sera, HCV RNA positive and negative strands were detectable in cells, which is compatible with the presence of replication. However, the viral load was small - positive HCV RNA strand was present at a concentration of 10(2) -10(3) per 10(6) cells, while the negative strand was ≤10(2) per 10(6) cells. We compared the RT activity of supernatant from day 3, 6, 9, and 12 for cells incubated with each of the 5 HCV-positive sera and each of the 5 HCV-negative sera (Figure 2A and 2B). Though there was sample-to-sample variation, the average of the RT activity from cells incubated with HCV-positive serum was higher than the average of the RT activity in cells incubated with HCV-negative serum at days 6, 9, and 12 post infection (Figure 2B). This could indicate that exposure to HCV-positive sera facilitates HIV replication; however, it could also indicate that sera from hepatitis C patients extend viability of HIV-infected cells. In support of the latter possibility came our observation that supernatant from cells infected with HIV and cultured with HCV-positive serum continued to demonstrate RT activity for 1-2 days longer than did the supernatant from cells infected with HIV only or with HIV followed by incubation with HCV-negative serum (data not shown).

To explore this possibility, we measured cell viability of HIV-infected cells incubated with HCV-positive patient serum and compared it to that of HIV-infected cells incubated with HCV-negative patient serum (Figure 2C). Cell viability was measured by staining cells with AnnexinV-FITC (AV) and propidium iodide (PI): viable cells were negative for both AV and PI. The results of this assay showed that cells incubated with either HCV-positive or HCV-negative serum with or without HIV infection remained similarly viable till Day 3. However, by day 5, only 20% of the HIV-infected cells incubated with HCV-negative serum remained viable, while 50% of the HIV-infected cells incubated with HCV-positive serum were still viable (Figure 2C). This suggested that the HCV-positive serum was inhibiting apoptosis in HIV-infected cells, and that this protection was not conferred by serum from healthy individuals.

**Figure 3:**
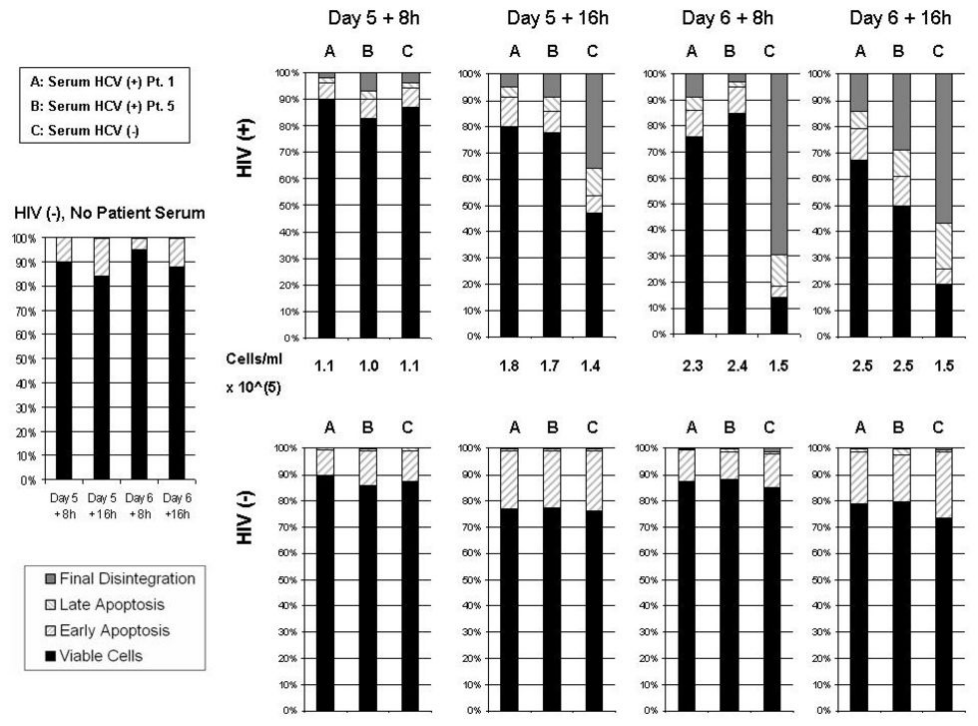
Time course of apoptosis in Daudi-CD4+ cells infected with HIV and incubated with either HCV-positive or HCV-negative patient serum. Daudi-CD4 cells were either HIV-infected (upper row) or mock HIV-infected (lower row) and incubated with either HCV-positive or HCV-negative patients’ sera. As additional control cells were mock HIV-infected and incubated with FBS only (no patient serum). Shown are the flow cytometry results from cells harvested at four different time points following HIV (or mock) infection: Day 5 plus 8 hours (128 hours), day 5 plus 16 hours (136 hours), day 6 plus 8 hours (152 hours), and day 6 plus 16 hours (160 hours). Each bar shows the percentage of cells in each of four phases: 1) viable: PI^-^, AV^-^, 2) early apoptosis: PI^-^, AV^+^, 3) late apoptosis: PI^+^, AV^+^, 4) final cell disintegration: PI^+^, AV^-^.

In the HIV-infected Daudi-CD4 cells, loss of viability had occurred rapidly during a 24-hour period between cell harvests; to track the changes in viability we assayed for apoptosis every 8 hours from Day 4 to Day 6 plus 16 hours (Figure 3 shows results from Day 5 + 8 hours to Day 6 + 16 hours). We tested effects of serum from two different HCV-positive patients (1W and 5W). To slow the rate of apoptosis, we reduced the infection dose of HIV to 2x10^4^ cpm and cell concentration was adjusted to 10^5^ per ml every 24 hours until day 5. This lower cell concentration was used to prevent early media depletion, which could affect cell viability. Uninfected cells that were not incubated with patient serum at all were viable throughout the experiment (Figure 3). Uninfected cells incubated with either HCV-positive or HCV-negative serum alone also remained 70-80% viable through Day 6 plus 16 hours. When cells were infected with HIV prior to incubation with patient serum, there was not only loss of viability, but there was also an increase in the number of cells in the “final” stage of apoptosis. The results in Figure 3 are nearly identical to what was seen in Figure 2C: HIV-infected cells incubated with HCV-positive serum 5W remained 50% viable, while HIV-infected cells incubated with HCV-negative serum were only about 20% viable. The results of HCV-positive serum 1W were even more dramatic in comparison to HCV-negative serum, with nearly 70% viability.

## Discussion

While the deleterious effects of HIV-coinfection on HCV-related liver disease are well documented, a number of studies suggest that HCV may also negatively affect the course of HIV disease. In particular, a report from the Swiss cohort study demonstrated that HIV/HCV coinfection was associated with increased likelihood of AIDS-defining events compared to infection with HIV alone [11]. Furthermore, CD4 cell recovery with HAART was slower in the presence of HCV infection. Similar finding were reported in large cohort studies from Italy and England [12,18]. A meta-analysis of 6216 patients from 8 trials confirmed that HAART-related immune reconstitution in HIV/HCV coinfected patients lags behind that in patients infected with HIV alone [19]. However, Sulkowski et al. [20] did not find any evidence that HCV infection substantially alters the risk of dying, developing AIDS, or responding immunologically to HAART. These discrepancies could be the result of differences among the studied populations and different prevalence of cofactors, such as IDU. However, it should be noted that no study so far has found a beneficial effect of HCV on HIV progression, although a related flavivirus - GB virus C/hepatitis G virus (GBV-C/HGV) - was credibly associated with better survival rates [21].

Our study provides evidence for the existence of possible biological basis of HCV-HIV interactions. HIV/HCV-coinfected patients demonstrated less CD4+ and CD8+ T cell apoptosis than patients infected only with HIV. This effect was largely abolished by standard anti-HCV treatment. Since interferon alpha demonstrates proapoptotic capabilities [22] it could have influenced these observations. However, it should be pointed out that the level of apoptosis correlated inversely with HCV RNA load in serum Furthermore, the dynamics of CD19+ B cells were reverse, as patients with double infection had higher apoptosis than HIV-monoinfected patients, and apoptosis decreased during antiviral treatment. The latter findings are compatible with those reported by Toubi et al, who found that spontaneous B cell apoptosis is increased in chronic hepatitis C but returns to normal values in sustained responders to antiviral treatment [23].

While HCV coinfection may inhibit HIV-related apoptosis, HIV/HCV co-infected patients demonstrate slower CD4+ cell recovery on HAART, than those infected with HIV only [11–13]. The reasons for this delayed CD4+ cell recovery are unclear, but could be related to HCV replication in lyphoid cells including CD4+ and CD34+ progenitor cells [24,25] or even to T-cells sequestration in the liver.

In contrast to our findings, two previous cross-sectional studies found increased lymphocyte apoptosis in HIV/HCV-coinfected patients when compared to HIV-monoinfected patients [26,27]. The reasons for this discrepancy are unclear; however, in these studies HIV/HCV-coinfected patients were overwhelmingly IDUs, whereas HIV-positive controls were not. Thus, it cannot be excluded that it was the drug use that affected the level of lymphocyte apoptosis. In contrast, while our HIV/HCV-coinfected patients were mostly IDUs, at least 6 months long abstinence was a prerequisite for inclusion into the study. Furthermore, the effect of HCV coinfection on apoptosis was suggested by longitudinal analysis of patients receiving antiviral therapy and by studies *in vitro* on cell line dually susceptible to HCV and HIV infections. While our *in vitro* cell system did not necessary mirror events occurring *in vivo*, there is evidence that there are some human cell types susceptible to infection by both viruses. In particular, native macrophages seem to be capable of supporting both infections and the same cell could harbor both pathogens, a least occasionally [9,28] However, the level of HCV replication is low, which is true for all extrahepatic sites and cell lines. Infection of monocytes/macrophages by HCV is not unexpected as these cells are known to be permissive to a wide range of viruses, including some other flaviviruses [29]

Apoptosis is considered to be the major mechanism of CD4+ T-cell depletion in HIV disease. There is convincing evidence that this are primarily the uninfected bystander cells that succumb to apoptosis, through a process that involves upregulation of death receptors and their ligands and downregulation of BCL-2 family survival factors [30,31]. Moreover, apoptosis may preferentially target HIV-1 specific CD4+ T cells, which further contributes to loss of immunological control of HIV-1 replication [32]. While uninfected T cells die by apoptosis, productively infected cells remain apoptosis resistant due to modulatory effects of various HIV proteins [30,33]. Several HIV gene products have been reported to have both anti-apoptotic and proapoptotic activities, presumably switching in response to the infection stage [31]. For example, Nef may prevent apoptosis of infected cell by downregulation of CD4 and MHC class I molecules, while it may also enhance apoptosis by upregulation of CD95-CD95L pathway and caspase activation. Inhibition of apoptosis may be dominant in the early phase of infection to enhance viral replication and infectivity [34].

It could be speculated that inhibition of “bystander” apoptosis by HCV coinfection would be beneficial in preserving the patient’s immune response; however, a similar effect on HIV-infected cells would be deleterious, as it would facilitate spread and replication of HIV. In our study exposure to HCV resulted in extended survival of HIV-infected Daudi cells and there was a parallel increase in HIV replication. Similarly, in a study done with Jurkat T cells engineered to express the adenovirus E1B 19K protein (a potent inhibitor of apoptosis), HIV persistence was enhanced due to increased cell viability [35].

The mechanism behind the inhibition of HIV-related apoptosis by HCV is unclear. Death-receptor-mediated apoptosis plays an important role in HCV-associated liver injury, and apoptosis of activated T-cells may contribute to chronic infection [36]. However, in conditional transgenic mice expressing core, E1, E2 and NS2 proteins, Fas-mediated apoptosis of liver cells and death were reduced, probably due to repressing the release of cytochrome c from mitochondria and suppressing caspase-9 and -3/7 activation [37]. HCV proteins may work in conjunction, as it was shown that HCV core-E1-E2 transgenic mice hepatocytes underwent less apoptosis than transgenic tissue expressing core only [38].

Another HCV gene product attributed with extensive modulatory effects is NS5A. This protein was shown to enhance antiapoptotic activities of the PI3K-AKT pathway through its interaction with p85 PI3K [39], and to inhibit apoptosis through binding to Bin1 [40] and p53 [41]. Recently, it was also found to suppress pro-apoptotic host cell K^+^ channel [42]. The issue of the effect of HCV infection on apoptosis is far from clear as various HCV proteins were attributed both pro- and anti-apoptotic effects when tested in cultured cells. These discrepancies could be the effect of different experimental conditions but also of the non-physiological overexpression of viral proteins in *in vitro* systems [36].

## Conclusions

We have found that HCV/HIV-coinfected patients have less T-cell apoptosis than patients infected with HIV alone. Likewise, HIV-infected Daudi B-cell line remained viable longer if exposed to HCV-positive serum. These results suggest that HCV is a molecular-level determinant in HIV disease.
